# Inhibition of autotaxin activity ameliorates neuropathic pain derived from lumbar spinal canal stenosis

**DOI:** 10.1038/s41598-021-83569-3

**Published:** 2021-02-17

**Authors:** Baasanjav Uranbileg, Nobuko Ito, Makoto Kurano, Kuniyuki Kano, Kanji Uchida, Masahiko Sumitani, Junken Aoki, Yutaka Yatomi

**Affiliations:** 1 Department of Clinical Laboratory Medicine, The University of Tokyo, Tokyo, Japan; 2 Department of Anesthesiology and Pain Relief Center, Graduate School of Medicine, The University of Tokyo, 7-3-1 Hongo, Bunkyo-ku, Tokyo, 113-8655 Japan; 3 Department of Health Chemistry, Graduate School of Pharmaceutical Sciences, The University of Tokyo, Tokyo, Japan; 4 Department of Pain and Palliative Medicine, The University of Tokyo Hospital, Tokyo, Japan

**Keywords:** Biochemistry, Neuroscience, Biomarkers, Diseases, Neurology

## Abstract

Lumbar spinal canal stenosis (LSS) or mechanical compression of dorsal root ganglion (DRG) is one of the causes of low back pain and neuropathic pain (NP). Lysophosphatidic acid (LPA) is a potent bioactive lipid mediator that is produced mainly from lysophosphatidylcholine (LPC) via autotaxin (ATX) and is known to induce NP via LPA_1_ receptor signaling in mice. Recently, we demonstrated that LPC and LPA were higher in cerebrospinal fluid (CSF) of patients with LSS. Based on the possible potential efficacy of the ATX inhibitor for NP treatment, we used an NP model with compression of DRG (CD model) and investigated LPA dynamics and whether ATX inhibition could ameliorate NP symptoms, using an orally available ATX inhibitor (ONO-8430506) at a dose of 30 mg/kg. In CD model, we observed increased LPC and LPA levels in CSF, and decreased threshold of the pain which were ameliorated by oral administration of the ATX inhibitor with decreased microglia and astrocyte populations at the site of the spinal dorsal horn projecting from injured DRG. These results suggested possible efficacy of ATX inhibitor for the treatment of NP caused by spinal nerve root compression and involvement of the ATX-LPA axis in the mechanism of NP induction.

## Introduction

Neuropathic pain (NP) is characterized by abnormal pain symptoms such as hyperalgesia and allodynia and is caused by damage to the peripheral or central nervous system^1,2^. NP also occurs as a secondary symptom in diseases including lumbar spinal canal stenosis (LSS), diabetes, cancer, and as a side effect of chemotherapy^3–5^. The underlying pathophysiology of NP still has not been well defined. Multiple mechanisms appear to be involved in the manifestation of the NP symptoms such as enhanced expression of Ca_v_α_2_δ, EphB1, and PKCγ in the DRG or dorsal horn, which are likely representative mechanisms for hyperalgesia^6–8^. In addition, demyelination and crosstalk among sensory fibers might underlie the mechanisms of allodynia^9,10^.

Among several lines of suggested mechanisms of NP, lysophosphatidic acid (LPA) is potentially a promising key molecule for NP development. LPA is a lysophospholipid produced mainly from lysophosphatidylcholine (LPC) by autotaxin (ATX)-lysophospholipase D^11^ and acts through defined G protein-coupled receptors (GPCR)^11–18^. Regarding the pathogenesis of pain, LPA reportedly activates peripheral nociceptor endings directly and indirectly by releasing the substance P from peripheral nerve endings^19,20^. Furthermore, LPA initiates NP^9,10,21^, which was demonstrated by intrathecal injection of the LPA. These actions of the LPA are implemented through LPA_1_ as deduced from receptor-deficient mice models or by using a short-lived LPA_1_ receptor antagonist^9,10,22^. In addition, administration of lysolecithin, i.e., LPC, induces demyelination, causing behavioral allodynia and hyperalgesia in rodents^23^.

Based on patient samples, we recently reported that the levels of LPA and LPC in the cerebrospinal fluid (CSF) are significantly associated with the clinical severity of NP^24^. We also confirmed that the LPA and LPC in CSF from patients with LSS correlated well with clinical manifestations and LPC species reflected those of the corresponding LPA species^25^. In an animal model of the spinal cord injury (SCI), increased levels of LPA were observed^26,27^ along with induction of the microglia/macrophage and demyelination in the spinal cord injury site. Also, the involvement of LPA/LPA_1_ signaling in NP through several cell types including microglia were studied in LPA_1_ conditional null mutant mice^28^.

This evidence indicates the involvement of LPA signaling in NP, raising the possibility that inhibition of LPA production, i.e., ATX inhibition, can relieve NP symptoms. Accordingly, we examined for the first time the effect of an ATX inhibitor on the progression of NP using a NP model with compression of DRG (CD model) in rats, which is considered as one of the models of lumbar spinal canal stenosis (LSS)^29^ and as a proxy for radicular pain in patients with LSS^30,31^. As an ATX inhibitor, we used a recently developed one, which has optimized enzymatic activity by carboxyl group incorporation and gained the novel, most potent and orally available compound ONO-8430506, among others^32^.

Using the rat CD model, we verified the effect of ATX inhibitor by examining the mechanical threshold of pain, LPA and LPC levels in the CSF, and microglia and astrocyte accumulation at the site of the spinal dorsal horn projecting from injured DRG.

## Results

### Modulation of ATX activity, LPC, and LPA levels in CSF and their corresponding correlations

**Figure 1:**
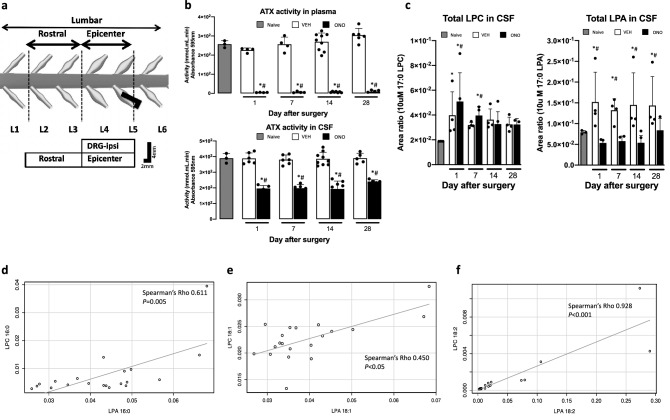
The CD model scheme and effect of the ATX inhibitor on production of the LPA in ONO group and corresponding correlations between LPC and LPA species in VEH group. (**a**) Schema of the CD model describing position and shape of the inserted stainless rod with tissue sampling information. (**b**) ATX activity measured in plasma (upper) and CSF (lower) of the naive, VEH and ONO groups at selected time points. ATX activity inhibited fully in plasma samples, whereas half of the activity remained in CSF of the ONO group at all selected time points. (**c**) Influence of the ATX inhibitor to the total LPC (left) and LPA levels (right) in CSF. LPC levels were increased in both group in day 1 and it was even more increased in the ONO group than VEH group showing interruption of the LPA biosynthesis from LPC. In the VEH group, LPA levels were significantly increased comparing with the ONO group at all the points. Values represent the mean ± SD (n = 5). Symbols indicate significant differences between groups analyzed by one-way ANOVA and paired t-test as follows: VEH, ONO groups with naive: p < 0.05*. VEH, ONO groups between each other at each selected time points: p < 0.05^#^. (**d**) Representative data of the correlation of the corresponding LPC and LPA species for the VEH group in CSF. LPC 16:0, 18:1, 18:2 was correlated with corresponding LPA 16:0, 18:1, 18:2 with Spearman Rho 0.611 (p = 0.005), 0.450 (p < 0.05) and 0.928 (p < 0.001) respectively. Values represent the mean ± SD (n = 3).

The ATX inhibitor ONO-8430506 efficiently inhibited the ATX activity and decreased LPA levels in the plasma when orally administered in rodents^32–35^. However, its effects on the ATX and LPA levels in CSF have not been tested. Therefore, we first examined the effect of ONO-8430506 administration on rat ATX activity and LPA levels in the CSF (Fig. 1). We did not show the LPA levels in the plasma and CSF with ATX inhibitor administration from normal rodent, because LPA level was too low similar to that of other study^34^. The ATX activity in the plasma and CSF did not change in the VEH (stainless steel rod inserted and control buffer administered) group. In the ONO (stainless steel rod inserted and ATX inhibitor administered) group, the ATX activity was partially (~ 50%) inhibited (Fig. 1b, lower), while it was fully inhibited in plasma (Fig. 1b, upper). In Fig. 1C, we depict total LPC levels (left) with enhanced changes in the VEH group after surgery on day 1 and more significant enhancement of total LPC in the ONO group at days 1 and 7. High levels of LPA was detected in the VEH groups from day 1 to 28. In contrast, in the ONO group, LPA levels were comparable to that of the naive group (Fig. 1c, right) on all days tested. Supplementary Fig. S1 shows detailed changes of each species of the LPC and LPA.

To investigate the origins of increased LPA in the VEH group, we analyzed correlations between fatty acid species of LPA and LPC. Significant correlations were observed in the VEH group between LPC and LPA species, especially between palmitic acid (16:0), oleic acid (18:1), and linoleic acid (18:2)-containing LPC and LPA (Fig. 1d–f). This result, together with the fact that ATX prefers these LPC species as its substrates, support the idea that ATX is responsible for LPA production in the CSF.

We further measured the mRNA expression levels of all six LPA receptors in the compressed L5 DRG on days 1, 7, 14, and 28, and we compared between VEH and ONO groups or to naïve group (Supplementary Fig. S2). LPA_1_, LPA_2_, LPA_4_, and LPA_6_ mRNA expression levels were significantly enhanced in the VEH group on day 28, but such increases were not observed in the ONO group.

Altogether, these results indicate that LPA signaling mediated by ATX and LPA receptors is enhanced in the NP model and that administration of ATX inhibitor is successful in suppressing LPA signaling by blocking the conversion of LPC to LPA in CSF.

### Prophylactic effect of an ATX inhibitor on NP

**Figure 2:**
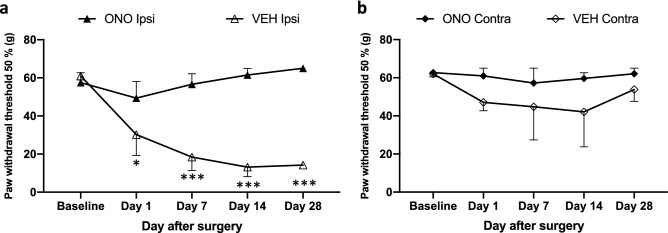
Therapeutic effect of the ATX inhibitor in CD model. Mechanical threshold of the pain measured in ipsilateral (**a**) and contralateral sides (**b**) of the plantar surface of the hind paws using von Frey filaments. Mechanical thresholds of the ipsilateral side were gradually recovered from mechanical pain by treatment of the ATX inhibitor in ONO group (closed triangle), showing significant changes in comparison to VEH group (open triangle). Mechanical threshold of the contralateral side gives no significant changes (closed and open diamonds). Values represent the mean ± SEM (n = 5). Symbols indicate significant differences between groups analyzed by two-way ANOVA with multiple comparison: VEH, ONO groups (ipsi- and contra-sides) between each other at each selected time points: p < 0.05*, p < 0.001***.

Mechanical pain threshold was measured in the ipsilateral and contralateral sides of the plantar surface of the hind paws in both the VEH and ONO groups, as shown in Fig. 2, and compared with baseline data (before surgery) from the same animal. We measured the threshold of pain until day 28 after surgery. In the VEH group (open triangle), mechanical pain thresholds of ipsilateral to L5 compression significantly decreased on day 1 and sustained to day 28. However, in the ONO group such decreases in the mechanical pain threshold were not observed throughout the entire period (closed triangle). It is noteworthy that in the ONO group, the threshold of pain was observed nearly identical to the levels of the baseline from day 7 and showed a significantly high threshold of the pain in comparison to the VEH group (Fig. 2a). There were no significant changes in the mechanical threshold of contralateral to L5 compression in either group (open or closed diamonds) (Fig. 2b).

### Effect of ATX inhibitor on early injury marker in CD model

**Figure 3:**
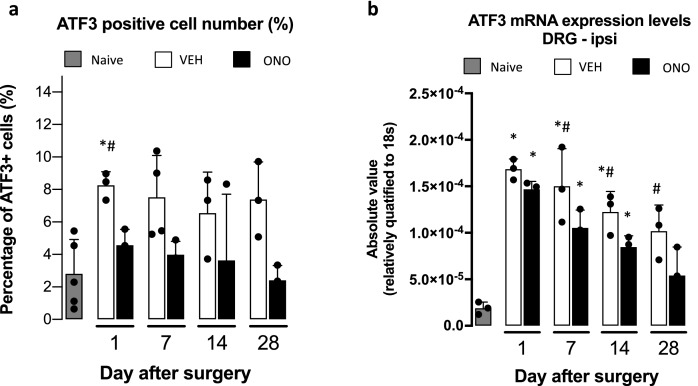
ATF3 expression pattern changes according to ATX inhibitor administration. Representative ATF3 expression levels depicted as percentage of ATF3 positive cells (**a**) or mRNA levels (**b**) in VEH and ONO groups at day 1, 7, 14 and 28 in comparison to naive group. Expression levels and positive stained cell numbers were decreased in ONO group by the effect of the ATX inhibitor administration. Values represent the mean ± SD (n = 3). Symbols indicate significant differences between groups analyzed by one-way ANOVA and paired t-test as follows: VEH, ONO groups with naive: p < 0.05*. VEH, ONO groups between each other at each selected time points: p < 0.05^#^.

We conducted an immunohistochemical analysis of the compressed L5 DRG in naive, VEH and ONO groups focusing on ATF3, which is a biomarker of tissue damage. ATF3-positive neurons were counted at different time points (days 1, 7, 14, and 28) and were significantly increased in the L5 ipsilateral DRGs in the VEH group on day 1, indicating acute injury (Fig. 3a) and representative IHC images shown in Supplementary Fig. S3. The number of ATF3-positive neurons in the ONO group was comparable to that in the naive group even at very early stage, suggesting the preventive effect of the ATX inhibitor on nerve injury. In DRG contralateral to nerve compression, we did not observe any changes in the total numbers of ATF3-positive neurons between the VEH and ONO groups (data not shown).

Increase in the level of ATF3 mRNA, which was prominently observed in the VEH group from day 1 to day 28, was again significantly suppressed in the ONO group (Fig. 3b).

### Effect of ATX inhibitor on increment of immune cells in the spinal cord

**Figure 4:**
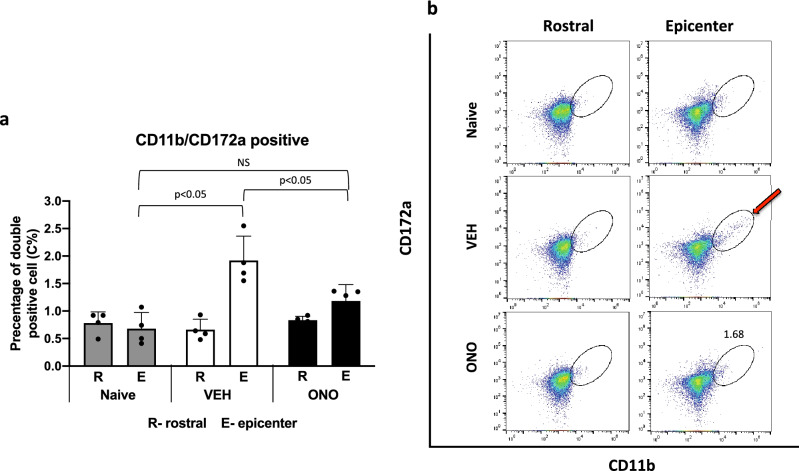
Flow cytometric analysis data of increased immune cells after modulation of the ATX activity. (**a**) CD11b/CD172A double-positive cells were counted and depicted as percentile from total cell number comparing epicenter (E) or rostral (R) sections of the spinal cord in naive, VEH and ONO groups at day 14 after model development. CD11b/CD172A double-positive cells were not different between groups in rostral section, whereas in epicenter section, significant (p < 0.05) increase of the cell numbers counted in the VEH group. In the ONO group double—positive cells did not differ from the naive group in epicenter (E) section. (**b**) Flow cytometric analysis and plot of elliptical area indicated the CD11b/CD172A double-positive cells. Arrow indicates increased immune cells (CD11b/CD172A double-positive) into the epicenter (E) section in the VEH group. In the ONO group amount of the immune cell were equivalent to that of naive group, showing the effect of the ATX inhibitor. Values represent the mean percentile ± SD (n = 3) and significant differences between groups analyzed by one-way ANOVA: VEH, ONO groups with naïve and paired t-test: VEH, ONO groups between each other.

We examined the effects of the ATX inhibitor on the increase of immune cells in the spinal cord by flow cytometric analysis at day 14 after the rod insertion. The rostral and epicenter sections of the spinal cord tissues, which are projected by the compressed L5 DRG were sampled in naive, VEH, and ONO groups. It is well known that microglia play important roles in the progression of NP after nerve injury, which has been explained not only by their activation but also by their accumulation at the injured site^36–38^. To quantitatively evaluate microglia accumulation, we counted CD11b/CD172A-double-positive cells using flow cytometry, since both markers are known to expressed by microglia (Fig. 4). In the VEH group, CD11b/CD172A positive cells significantly increased in number in the epicenter section (Fig. 4b). In the ONO group, the numbers of (by percentage) CD11b/CD172A positive cells were significantly lower than in the VEH group, comparable to the naive groups (Fig. 4b). In contrast, numbers of CD11b/CD172A positive cells in the rostral spinal cord tissues were comparable among the three groups. Figure 4b shows representative results from flow cytometry in the form of scatter plots of the CD11b/CD172a double-positive cell population. These results indicated that the ATX inhibitor inhibits the increase of microglia in the spinal cord near the site of mechanical compression of the DRG.

### Effect of ATX inhibitor on microglia

**Figure 5:**
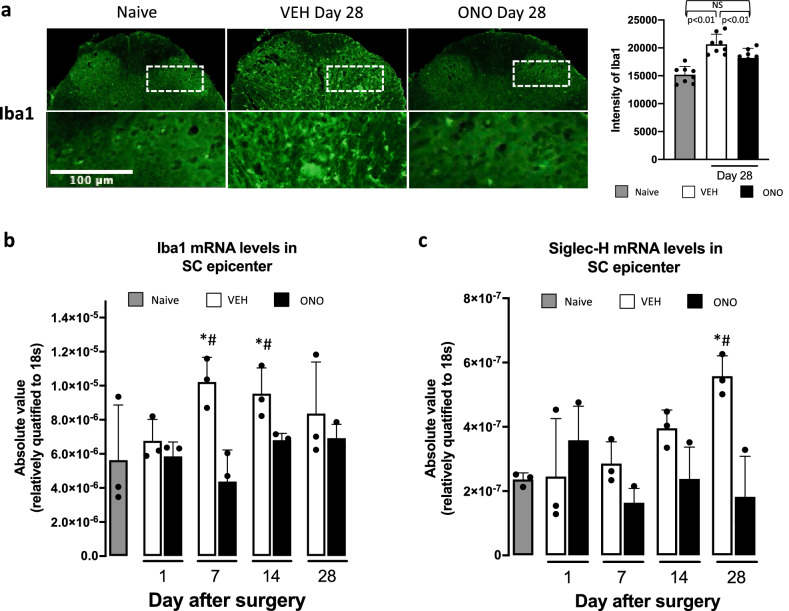
Inhibition of LPA biosynthesis declines an increase of the microglia in injured spinal cord. (**a**) Results represent images of the immunohistochemistry data for single staining of the Iba1 in the dorsal horn at day 28 after model development and their fluorescence intensity of Iba1 in naive, VEH and ONO groups. The fluorescence intensity of Iba1 significantly increased in VEH group in comparison to naïve, whereas in the ONO group, intensity of Iba1 was significantly lower than that of VEH group. Values represent the mean ± SD (n = 8) and significant differences between groups analyzed by one-way ANOVA with Bonferroni correction. The mRNA expression levels of Iba1 (**b**) and Sighlec-H (**c**) in epicenter sections of the spinal cord tissue samples in VEH and ONO groups in comparison to naive at selected time points. Significant enhancement of the Iba1 and Siglec-H expression levels were detected in the VEH group at days 7, 14 and at day 28, respectively. In the ONO group, there were no changes in expression levels of Iba1 and Siglec-H. Values represent the mean ± SD (n = 3). Scales: 100 μm. Symbols indicate significant differences between groups analyzed by one-way ANOVA and paired t-test as follows: VEH, ONO groups with naive: p < 0.05*. VEH, ONO groups between each other at each selected time points: p < 0.05^#^.

To confirm the increment of microglia at the site of injury, we examined the expression of Iba1, a marker of microglia, between VEH and ONO groups using anti-Iba1 antibody (Fig. 5a). At 28 days after surgery, the Iba1-stained cells significantly increased in the L5-level spinal cord dorsal horn of the epicenter section in the VEH but not in the naive group. Accumulation of Iba1-positive cells was not observed in the ONO group. Results of the fluorescence intensity, measured in four different tissue sections of the same samples in dorsal horn and at least three different animals in respective groups, are demonstrated in a bar graph with statistically significant changes (significance confirmed by the Bonferroni correction). We also examined Iba1 expression at the mRNA level using RT-PCR (Fig. 5b). In the VEH group, high expression of Iba1 mRNA was detected, especially on days 7 and 14, which was found to be significantly lower in the ONO group, similar to the naive group. As Iba1 is expressed not only by microglia but also by macrophages, we also determined mRNA expression levels of Siglec-H (Sialic-acid binding Immunoglobulin-like lectin-H), an authentic marker of microglia^39,40^. Unlike Iba1, Siglec-H mRNA gradually increased with time, with the highest level on day 28 in the VEH group. Of note, at this time point, Siglec-H mRNA levels in the VEH group were significantly higher than those of the ONO group (Fig. 5c).

### Effect of ATX inhibitor on astrocytes

**Figure 6:**
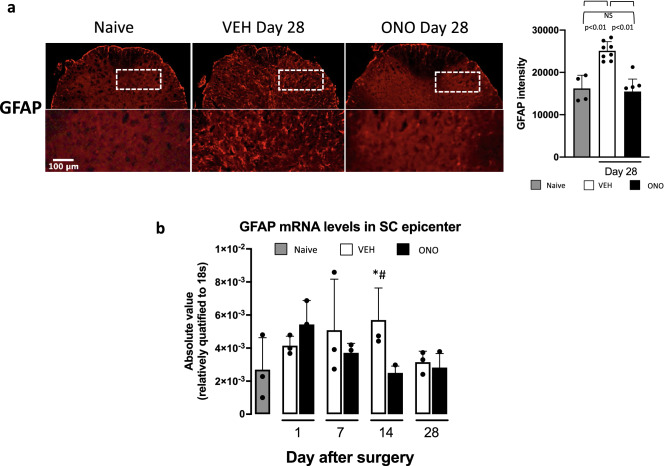
Astrocytes increment and activation were responded to low levels of the LPA in the ONO group. (**a**) The immunohistochemistry of GFAP were performed in the same tissue samples used for Iba1 staining and the fluorescence intensity of GFAP were analyzed. Fluorescence intensity of GFAP were increased in the VEH group significantly at day 28 compared with naive group, and it was decreased in the ONO group significantly compared with VEH group. Values represent the mean ± SD (n = 4–8) and significant differences between groups analyzed by one-way ANOVA with Bonferroni correction. (**b**) The mRNA levels of the GFAP were increased in the VEH group significantly at day 14 compared with naive, and in ONO group it was decreased to the same levels as in naive. Values represent the mean ± SD (n = 3). Scales: 100 μm. Symbols indicate significant differences between groups analyzed by one-way ANOVA and paired t-test as follows: VEH, ONO groups with naive: p < 0.05*. VEH, ONO groups between each other at each selected time points: p < 0.05^#^.

Based on the evidence that activated microglia release cytokines, which in turn activate nearby astrocytes^41,42^, we also examined the astrocytes in the epicenter spinal cord tissue in this CD model on day 28 and the effect of the ATX inhibitor. The serial sections shown in Fig. 5 were stained with antibodies against GFAP, a marker of astrocytes and the fluorescent intensities were determined (Fig. 6a). Similar to microglia (Fig. 5), a number of cells in the VEH group were found to be strongly stained with anti-GFAP. The mRNA levels of GFAP over time as judged by RT-PCR were also suppressed in the ONO group at day 14. In the VEH group, GFAP mRNA expression levels increased gradually from the very acute phase to 14 days, but at day 28 they recovered to their naive levels. In the ONO group, the mRNA expression levels of GFAP tended to increase at the very acute phase of DRG compression, and gradually decreased and at day 14 recovered to normal levels comparable to those in the naive group (Fig. 6b).

## Discussion

The preventive effect of the ATX inhibitor ONO-8430506 was demonstrated in a rat NP model of the LSS through inhibition of LPA production. In the process of NP formation, ATF3 induction in the DRG is the initial cellular response from injured neurons^43^, followed by accumulation of microglia and astrocytes. Activated microglia are known to cause temporal activation of various enzymes and pro-inflammatory cytokine release, which in turn activate nearby astrocytes, and then the microglia and astrocytes contribute to maintenance of NP symptoms^38,44–47^. Activated astrocytes also release cytokines and activate nearby cells such as neurons and microglia^41,42^. LPA has been reported as the key signal that initiates glial activation^47,48^. Treatment with the ATX inhibitor prevented the steps of NP formation and consistently maintained low levels of LPA in the CSF throughout the study period. These results demonstrated that LPA is strongly involved in NP generation and maintenance during nerve compression related to LSS.

In our recent studies, we have confirmed increased levels of LPC and LPA in CSF, plasma, and spinal cord tissue samples in the central type of LSS with NP model^27^. Furthermore, these findings were replicated in CSF samples of LSS patients with NP^24,25^. Similarly, in this study LPC levels of CSF at day 1 and LPA levels of CSF at all experimental periods were also clearly enhanced in the VEH group, revealing that increases in LPC and LPA in CSF are strongly involved in the mechanism of LSS with NP. All 12 molecular species of LPC and 11 corresponding LPA species, except for LPA 22:5, were measured (Supplementary Fig. S1). The corresponding LPC and LPA species were strongly correlated with each other, indicating that each LPA species was converted from the corresponding LPC species with the action of ATX, which exists abundantly in extracellular space, such as CSF^49^. There might be another possibility of LPA being produced intracellularly by hydrolysis of PA (phosphatidic acid) by PLA_1_ and PLA_2_. Considering that LPA levels in the CSF were dramatically high and suppression of these levels by the ATX inhibitor coincides with the recovery of pain threshold, it is appropriate to conclude that LPA synthesis in extracellular space would lead to a more efficient supply of LPA receptors on the surface of neurons and microglia. In addition to LPA levels in the CSF, increased mRNA expression levels of the LPA receptors were also decreased by the ATX inhibitor, indicating its effect not only on ligand production but also on receptor expression (Supplementary Fig. S2).

ATX has been detected in normal CSF in abundance and LPA is produced from LPC by ATX in CSF constantly^49^. Therefore, increased local production of LPC in compressed tissue contributes to substrate supply for further LPA production^27^. Abundantly existing ATX in CSF might fail to detect the precise increases of LPC, and treatment of the ATX inhibitor probably resulted in both an increase in LPC levels and a decrease in LPA levels. LPC itself has also been reported as an inducer of demyelination during NP^23,50^, but only at high doses. There were huge differences in the LPA levels, but not in LPC levels, of the CSF between the ONO group and VEH group in accordance with the differences in the mechanical threshold. These results suggest that LPA itself is much more important than LPC, consistent with previous reports^51,52^. An increase in LPA synergistically leads to cellular activation of the neuron, microglia, and astrocytes as they play key roles in the progress and maintenance of LSS with NP^9,10,22,53^.

Considering previous notions, the possible mechanism for improvement of NP would be the inhibition of the initial response of DRG neurons, increment of microglia and astrocytes in the spinal cord near the site of the mechanical compression of the DRG. In the present study, we confirmed the involvement of the microglia and astrocytes in the CD model by performing FACS, immunohistochemistry, and measuring expression of the mRNA levels of the microglia and astrocyte markers. Similar to previous reports^28,54–56^, we obtained increase of microglia and astrocytes in the injured site, and inhibition of ATX could interrupt these increases. These results suggest the involvement of the LPA in the accumulation and activation of microglia and astrocytes in NP at injured nerve sites. Apart from these glial cells, we should consider the alternative mechanism involving the initial neuronal responses by LPA. Since direct activation of TRPV1in primary neurons of the DRG can induce NP^16,57^, decreased LPA levels by inhibition of ATX could ameliorate NP in this study.

There are several lines of data showing the possible usefulness of the ATX inhibitory agents for the treatment of various diseases caused by LPA, including breast and thyroid cancer, and urethral tension^32–35,58,59^. With sufficient inhibition of the ATX activity, these treatments generally do not affect the general conditions of the experimental animals. Here, we revealed that the ATX inhibitor fully inhibited the ATX activity in the plasma, but some residual activity was observed in the CSF. Such differences might presume the involvement of the blood–brain-barrier because of the oral administration of the ATX inhibitor. Therefore, the ATX inhibitor used in the present study would have little effect on general conditions and reflect the analgesic potency of the NP caused by LSS.

In clinical practice, pregabalin and its related compounds are effective at present for the treatment of NP associated with post-herpetic neuralgia and diabetic neuropathy in addition to epilepsy treatment^60–63^. The necessity of new drugs for NP based on its underlying mechanisms is anticipated. The promising therapeutic effect of the ATX inhibitor in NP is described herein, in addition to a previous study where an LPA_1_ antagonist was used^22^, which indicates the importance of ATX/LPA/LPA_1_ signaling in NP.

A limitation of this study should also be considered. We performed all studies only in female rats with established concentrations of the ATX inhibitor. Additional detailed studies should be conducted to determine the core mechanisms behind microglia and astrocyte up-regulation, involvement in the maintenance of NP, focusing on gender-distinct experimental rodents together with variable concentrations of the ATX inhibitor. In addition, more accurate studies needed regarding involvement of other LPA receptor subtypes in ATX/LPA/LPA signaling such as LPA_3_ which was previously indicated the activation of microglia and astrocyte^64^.

In conclusion, using the CD model, we observed a decreased threshold of pain, which was detected in behavioral studies as NP symptoms. Increased LPC and LPA levels in CSF suggested the involvement of the ATX-LPA pathway as one of the main mechanisms. To our knowledge, the current study is the first report to show the importance of oral administration of the ATX inhibitor, which could disrupt LPA production from LPC, and successfully increase the threshold of pain and significantly decreased the population of activated DRG neurons, accumulation of microglia, and astrocyte cell population at the site of the injury. This would help focus on a new target to treat NP, which remains intractable in current clinical settings.

## Methods

### Animals

8–10 weeks old adult female Sprague–Dawley rats (200–250 g) obtained from Japan SLC (Shizuoka, Japan) were used in this study. The rats were housed in a 12 h light/12 h dark cycle with ad libitum access to food and water. All the animal experiments were conducted in accordance with the guidelines for the Care and Use of Laboratory Animals and were approved by the Ethic Committee for Animal Experiment of the University of Tokyo (approval No. P15-100). All the animal studies were carried out in compliance with the ARRIVE guidelines.

### Surgical procedure

Mechanical compression of the DRG (CD model) was generated using the method as described here^29,65,66^. Briefly, animals were anesthetized by intraperitoneal injection of the pentobarbital (6.5 mg/kg) (Kyoritsu Seiyaku, Tokyo, Japan) and inhalation of isoflurane (Abbott, Illinois, USA), while in a prone position. All surgical procedures were performed under the Leica M80 stereomicroscope (Leica Microsystems, Buffalo Grove, IL, USA). A skin incision was made over the spinal midline, and the paraspinal muscles were separated from the spinal processes at the left L5 level to expose the intervertebral foramina. An L-shaped stainless steel rod (Ø 1 mm) (Daidohant, Osaka, Japan) was inserted to compress the DRG at the left L5 position, and the incision was closed using a sterile needle and disposable skin stapler (Smith & Nephew, London, UK). Sham-operated animals underwent a similar surgical procedure except for the L-shaped stainless steel rod insertion. Rats were returned to the cage after being placed on a 37 °C heating blanket for 1 h.

The schema of the spinal cord and the position of the inserted stainless rod is depicted in Fig. 1A, together with the spinal cord and DRG sampling group.

### Behavioral testing

The rat mechanical stimulus response using the von Frey filament test (North Coast, Medical and Rehabilitation products, Morgan-Hill, CA, USA) was collected 3 days before surgery as a basement and on days 1, 7, 14, and 28 post-surgery. The behavioral testers were blinded during testing about groups with ATX inhibitor or control buffer administration and the same two testers performed the von Frey test throughout the study. Rats were placed in nontransparent plastic cubicles on a special mesh-floored cage in a soundproof room for 30–60 min to acclimate and the plantar surface of the hind paws were stimulated by using different-sized von Frey filaments (4.31, 4.74, 5.07, 5.46, 5.88, 6.1) threaded under the mesh floor. The stimulation was performed by placing the filament perpendicularly to the plantar surface for 6 s. The responses were considered to be positive when the rat escaped the stimulated leg on application of the filaments. In the absence of a response, 60 g was considered as the cut-off point. The 50% paw withdrawal threshold (PWT) was determined using the up-down method^67^.

### ONO-8430506 preparation and administration

ONO-8430506 was obtained from Ono Pharmaceuticals (Osaka, Japan; patent no. WO2012005227) under a material transfer agreement. It was prepared at a concentration of 3 mg/mL in 25 mM sodium hydroxide and heated to 60 °C for five min to dissolve. All solutions were filtered through a 0.22-µm filter before administration. Control 25 mM sodium hydroxide solution or 30 mg/kg ONO-8430506 was orally administered once a day or starting two days before surgery and continued until selected time points, respectively. No other medications administered pre or post-surgery.

### Immunohistochemistry

For IHC, rat tissue samples were prepared as described below^68^. In addition to spinal cord tissues from the compressed level, DRGs from left and right L5 levels were removed and fixed in 4% paraformaldehyde phosphate buffered solution (Wako laboratory chemicals, Osaka, Japan) and dehydrated in 15–30% sucrose. Frozen sections were cut using a cryostat (Leica CM1850, Wetzlar, Germany) at 8–10 µm thickness and mounted on glass slides. Sections were immunostained with a mouse monoclonal antibody for ATF3 (1:300, Santa Cruz Biotechnology, Texas, USA), rabbit polyclonal Iba1 (1:200, Wako Chemicals, Osaka, Japan) and glial fibrillary acidic protein (GFAP) (1:500, Cell Signaling Technology, Danvers, Massachusetts, USA) as a primary, and goat anti-mouse Alexa Fluor 594 and goat anti-rabbit Alexa Fluor 488 (1:500, Thermo Fisher Scientific, Massachusetts, USA), as a secondary antibody. Images were developed using a BZ-X700 All-in-one Fluorescence Microscope from KEYENCE Corporation (Osaka, Japan). For the quantification of fluorescence intensity, an image analyzer, Fiji Image J (NIH Image) was used.

### Quantitative real-time polymerase chain reaction

The total RNA of the spinal cord tissues and DRGs from the stainless steel rod inserted or control levels were extracted using the GenElute mammalian total RNA miniprep kit (Sigma Aldrich, St Louis County, Missouri, USA). One microgram of purified total RNA was transcribed using a SuperScript™ First-Strand Synthesis System for RT-PCR (Roche Molecular Diagnostics, CA, USA). Quantitative real-time PCR was performed with a TaqMan Universal Master Mix (Applied Biosystems, Life Technologies, CA, USA) using a 7300 Real Time PCR System (Applied Biosystems). ATF3, *Lpar1, Lpar2, Lpar3, Lpar4, Lpar5, Lpar6,* ATX, Iba1, Siglec-H, GFAP, and internal control 18s ribosomal primers and probes (TaqMan Gene Expression Assays) were obtained from Applied Biosystems (Rn00563784_m1, Rn00588435_m1, Rn01420531_m1, Rn00576734_m1, Rn03037115_s1, Rn02758966_s1, Rn03415828_s1, Rn01505088_m1, Rn00574125_g1, Rn0140895_m1, Rn01253033_m1 and HS99999901_s1). The samples were incubated for 10 min at 95 °C, followed by 40 cycles at 95 °C for 15 s and 60 °C for one min. The target gene mRNA expression level was quantified relative to ribosomal 18s using the 2-ΔΔCt method (Applied Biosystems, User Bulletin No 2).

### Flow cytometry analysis

At day 14 after surgery rats were deeply anesthetized and peripheral blood was completely removed by transcardial perfusion with phosphate buffered saline (PBS). The spinal cord (5 mm) from the compressed or uncompressed (control) L5 levels were carefully dissected from the vertebral column and mechanically dissociated in 250 U/mL collagenase (Sigma Aldrich, Missouri, USA) containing HBSS (Gibco, San Diego, CA, USA). The mixtures were incubated at 37 °C for 1 h, then passed through a 70 µm nylon cell strainer (Ref 352350, Falcon, Corning, NY, USA), cells were washed in Dulbecco’s Modified Eagle Medium (DMEM) containing 10% fetal bovine serum, and centrifuged at 300×*g* for five min at 4 °C. The pellets were re-suspended in 2 mL stain buffer (Cat: 554656, BD Biosciences, USA), using cell strainer-containing tubes (Ref 352235), centrifuged, and followed by a 15 min incubation on ice with Fc Block (FcR Blocking Reagent, MACS, Myltenyi Biotec) and an additional incubation on ice for 30 min with fluorescent antibodies. Samples were stained with the following antibodies: FITC mouse IgG1 (400108 BioLegend, San Diego, CA, USA), APC Mouse IgA (562140 BD Biosciences) for control samples, and FITC anti-rat CD45 (202205, BioLegend), APC Mouse anti-rat CD11b (562102 BD Biosciences), and anti-rat CD172a (SIRPa-APC, 130-107, MACS, Miltenyi Biotec) for macrophage/microglial cells. Before analysis, propidium iodide was added to determine the cell viability. All samples were suspended and analyzed at the same flow rate and duration using an Accuri C6 Flow Cytometer (BD Biosciences). The data were analyzed using FlowJo software (FlowJo LLC, USA).

### Collection of the CSF and plasma for lysophospholipid measurements

#### CSF collection

CSF was collected from the cisterna magna as described^69^ with minor modifications using stereomicroscopy. Animals were anesthetized by inhalation of isoflurane, which is connected to a stereotaxic device (David Kopf instrument, Tujunga, California, USA) where the rats were mounted. Skin incision was made, and superficial muscles were dissected using bipolar forceps (Protech International Inc., San Diego, CA, USA). After the retractor was placed, the underlying layer of muscles were separated carefully to avoid accidental puncture of the dura. When the dura was exposed, the CSF was collected using 30 G 1/2 needles (Dentronics, Tokyo, Japan) connected to a 1 mL syringe. Collected samples (50–120 µL) were labeled and stored at − 80 °C.

#### Plasma collection

Blood samples were collected from the jugular vein and treated with ethylene-diamine-tetra-acetic acid and citrate-theophylline-adenosine-dipyridamole (BD Biosciences, Tokyo, Japan). The samples were centrifuged at 2500×*g* for 30 min at 4 °C, and the plasmas obtained were stored at − 80 °C until lysophospholipid measurement^70^.

### Measurements of lysophospholipids (LysoPLs) in rat CSF samples

The LysoPLs were quantified using LC–MS/MS, as previously described^71^. Briefly, the CSF samples were mixed and sonicated with methanol and an internal standard (1 µM LPA 17:0 or 10 µM LPC 17:0). After centrifugation at 21,500 g, the resulting supernatant was transferred to a sample tube for LC–MS/MS analysis. Then, the methanol extract (20 µL) was analyzed in an autosampler (Nanospace LC, Shiseido) equipped with a C18 CAPCELL PAK ACR column (1.5 × 250 mm; Shiseido) and LysoPLs were extracted using a gradient of solvent A (5 mM ammonium formate in water) and solvent B (5 mM ammonium formate in 95% (v/v) acetonitrile). The eluate was sequentially ionized with electrospray ionization using a Quantum Ultra triple quadrupole mass spectrometer (Thermo Fisher Scientific). For each LysoPL class, 12 acyl chains (14:0, 16:0, 16:1, 18:0, 18:1, 18:2, 18:3, 20:3, 20:4, 20:5, 22:5, and 22:6) were monitored in both positive and negative ion modes. The concentrations of LysoPLs were calculated from the area ratio to the internal standard: LPA 17:0 (for LPA, LPE, LPI, LPG, and LysoPS species) or LysoPC 17:0 (for LPC species).

### Measurement of the LysoPLD activity in rat CSF and plasma samples

LysoPLD activity was assessed based on the amount of choline released with the use of LPC as the substrate, as described^72^: the reactions were performed in 100-µL aliquots. Serum samples (20 µL) and CSF (10 µL) were incubated with 2 mM 1-myristoyl (14:0)–LPC (Avanti Polar Lipids Inc., Alabaster, AL) in the presence of 100 mM Tris–HCl, pH 9.0, 500 mM NaCl, 5 mM MgCl2, 5 mM CaCl2, and 0.05% Triton X-100 for 3 h at 37 °C. The liberated choline was detected by an enzymatic photometric method using choline oxidase (Asahi Chemical, Tokyo, Japan), horseradish peroxidase (Toyobo, Osaka, Japan), and TOOS reagent (N-ethyl-N-(2-hydoroxy-3-sulfoproryl)-3-methylaniline; Dojindo Molecular Technologies, Inc. Tokyo, Japan) as a hydrogen donor. This choline measurement was performed using an absorption spectrometer (Infinite F50, TECAN, Zurich, Switzerland).

### Statistical analysis

Data processing and analysis were performed using R statistical software version 3.3.1 (http://www.r-project.org), and data were processed with Graphpad Prism 8.0 software (GraphPad Software, San Diego, CA). Behavioral test results were analyzed by two-way repeated measure ANOVA with multiple comparison and to show significance effectively we used SEM only here. A paired t-test, one-way ANOVA were used to analyze differences in the mRNA levels of all measured markers, fluorescence intensity and the levels of LPL species in CSF, plasma, and tissue samples of the spinal cord DRG. The results of the LPL species are expressed as the means and standard deviations (SD). The correlations between LPL species were analyzed by non-parametric measure of the Spearman’s rank-order correlation. The results were considered significant when P-values were < 0.05 (for fluorescence intensity the significance confirmed by the Bonferroni correction).

## Supplementary Information

Supplementary Information.
