# Supplementary material for: Inhibition of autotaxin activity ameliorates neuropathic pain derived from lumbar spinal canal stenosis
Source: Sci Rep. 2021 Feb 17;11:3984. doi: 10.1038/s41598-021-83569-3 (PMC7889906; doi:10.1038/s41598-021-83569-3)
Supplement: Supplementary file 1 — Supplementary Information. [file 41598_2021_83569_MOESM1_ESM.pdf]

Inhibition of autotaxin activity ameliorates neuropathic pain derived from lumbar spinal stenosis

Baasanjav Uranbileg[1], Nobuko Ito[2]*, Makoto Kurano[1], Kuniyuki Kano[3], Kanji Uchida[2], Masahiko Sumitani[4], Junken Aoki[3] and Yutaka Yatomi[1]

[1] Department of Clinical Laboratory Medicine, The University of Tokyo, Tokyo, Japan
[2] Department of Anesthesiology and Pain Relief Center, The University of Tokyo, Tokyo, Japan
[3] Department of Health Chemistry, Graduate School of Pharmaceutical Sciences, The University of Tokyo, Tokyo, Japan
[4] Department of Pain and Palliative Medicine, The University of Tokyo Hospital, Tokyo, Japan

* Corresponding author:
Nobuko Ito, M.D., Ph.D.
Department of Anesthesiology and Pain Relief Center
Graduate School of Medicine, The University of Tokyo
7-3-1 Hongo, Bunkyo-ku, Tokyo 113-8655, JAPAN
Phone: +81-3-3815-5411
Fax: +81-3-5800-8938
E-mail: nobuko-tky@umin.ac.jp

Supplementary figure S1

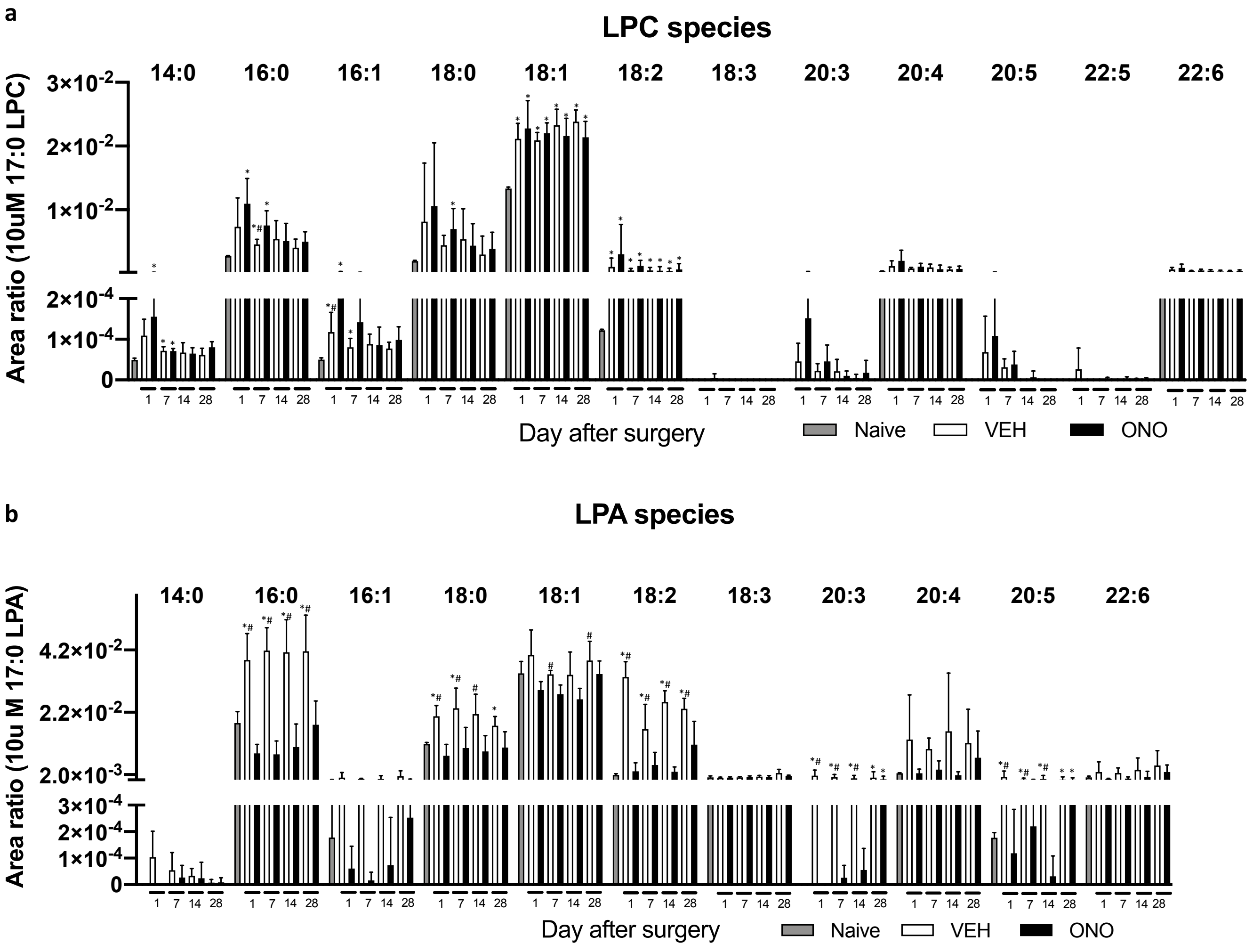

Supplementary Figure S1. LPC and LPA species in CSF. a. All 12 molecular species of LPC were measured and significant increased levels in VEH group observed mainly in 16:1, 18:1, 18:2 species and more significant enhancement in the ONO group. b. 11 molecular species of LPA except 22:5 was measured, and high levels of the LPA were detected in 16:0, 18:0, 18:2, 20:3, 20:5 in the VEH group. In ONO group LPA levels returned to the level of the naïve group.

Symbols indicate significant differences between groups analyzed by one-way ANOVA and paired t-test as follows: VEH, ONO groups with naive: p<0.05*. VEH, ONO groups between each other at each selected time points: p<0.05#.

Supplementary figure S2

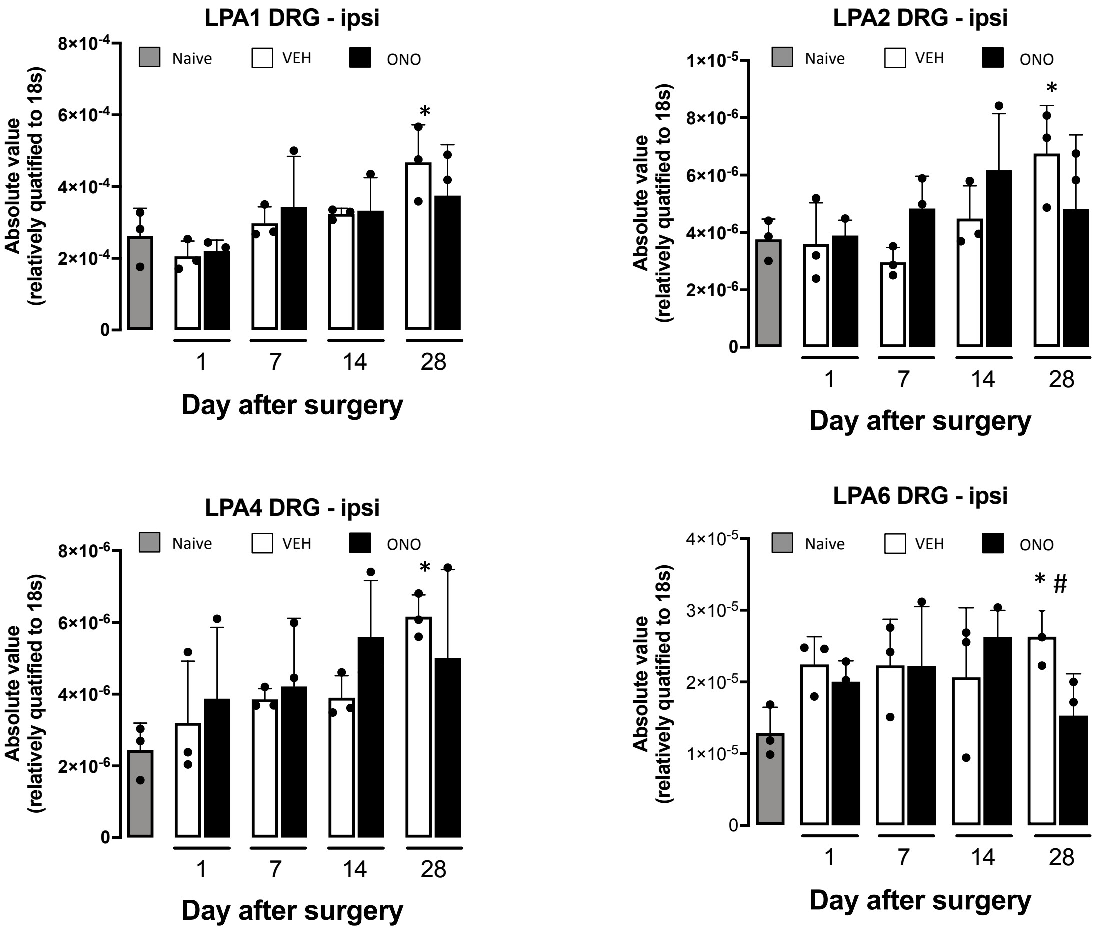

Supplementary Figure S2. The mRNA expression levels of the LPA receptors in left L5 DRG tissue samples from naive, VEH and ONO groups. There were no significant changes in expression levels of the LPA1, LPA2, LPA4, and LPA6 until day 28 after surgery in both groups. In the VEH group on day 28 significant increased levels were observed in LPA1, LPA2, LPA4 in comparison to naive and LPA6 in comparison to naive and ONO group. Absolute values of the mRNA levels were calculated. Values represent the mean ± SD (n=3).

Symbols indicate significant differences between groups analyzed by one-way ANOVA and paired t-test as follows: VEH, ONO groups with naive: p<0.05*. VEH, ONO groups between each other at each selected time points: p<0.05#.

Supplementary figure S3

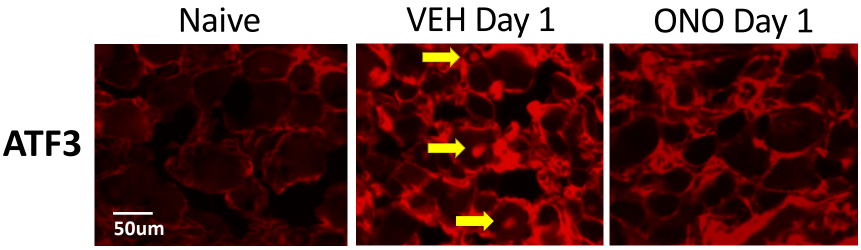

Supplementary Figure S3. The immunohistochemistry of the early injury marker ATF3 expression. Representative ATF3 expression levels depicted as immunohistochemistry analysis of left L5 DRG tissue samples for day 1. Arrow indicated positive signal of ATF3 in nucleus of the VEH group. Positively stained cells in ONO group were similar to naive by the effect of the ATX inhibitor administration. Scales: 50μm.
